# Induction of tumour-specific CD8^+^ cytotoxic T lymphocytes by tumour lysate-pulsed autologous dendritic cells in patients with uterine serous papillary cancer

**DOI:** 10.1038/sj.bjc.6600026

**Published:** 2002-01-07

**Authors:** A D Santin, S Bellone, A Ravaggi, J J Roman, S Pecorelli, G P Parham, M J Cannon

**Affiliations:** Department of Obstetrics and Gynecology, UAMS Medical Center, Division of Gynecologic Oncology, University of Arkansas, 4301 W. Markham, Little Rock, Arkansas AR 72205-7199, USA; Department of Microbiology and Immunology, University of Arkansas 4301 W. Markham, Little Rock, Arkansas AR 72205-7199, USA; Division of Gynecologic Oncology, University of Brescia, Brescia, Italy

**Keywords:** serous papillary uterine cancer, CTLs, dendritic cells, tumour lysate

## Abstract

Uterine serous papillary carcinoma is a highly aggressive variant of endometrial cancer histologically similar to high grade ovarian cancer. Unlike ovarian cancer, however, it is a chemoresistant disease from onset, with responses to combined cisplatinum-based chemotherapy in the order of 20% and an extremely poor prognosis. In this study, we demonstrate that tumour lysate-pulsed autologous dendritic cells can elicit a specific CD8^+^ cytotoxic T lymphocyte response against autologous tumour target cells in three patients with uterine serous papillary cancer. CTL from patients 1 and 2 expressed strong cytolytic activity against autologous tumour cells, did not lyse autologous lymphoblasts or autologous EBV-transformed cell lines, and were variably cytotoxic against the NK-sensitive cell line K-562. Patient 3 CD8^+^ T cells expressed a modest but reproducible cytotoxicity against autologous tumour cells only at the time of the first priming. Further priming attempts with PBL collected from patient 3 after tumour progression in the lumboaortic lymph nodes were unsuccesful. Cytotoxicity against autologous tumour cells could be significantly inhibited by anti-HLA class I (W6/32) and anti-LFA-1 MAbs. Highly cytotoxic CD8^+^ T cells from patients 1 and 2 showed a heterogeneous CD56 expression while CD56 was not expressed by non-cytotoxic CD8^+^ T cells from patient 3. Using two colour flow cytometric analysis of intracellular cytokine expression at the single cell level, a striking dominance of IFN-γ expressors was detectable in CTL populations of patients 1 and 2 while in patient 3 a dominant population of CD8^+^ T cells expressing IL-4 and IL-10 was consistently detected. Taken together, these data demonstrate that tumour lysate-pulsed DC can be an effective tool in inducing uterine serous papillary cancer-specific CD8^+^ CTL able to kill autologous tumour cells *in vitro*. However, high levels of tumour specific tolerance in some patients may impose a significant barrier to therapeutic vaccination. These results may have important implications for the treatment in the adjuvant setting of uterine serous papillary cancer patients with active or adoptive immunotherapy.

*British Journal of Cancer* (2002) **86**, 151–157. DOI: 10.1038/sj/bjc/6600026 www.bjcancer.com

© 2002 The Cancer Research Campaign

## 

Uterine serous papillary carcinoma (USPC) is a histologic subtype of endometrial cancer constituting up to 10% of all endometrial cancers. Histologically similar to high grade ovarian cancer (Carcangiu and Chambers, 1992; Sherman *et al*, 1992), USPC has a propensity for early intra-abdominal and lymphatic spread even at presentation (Carcangiu and Chambers, 1995; Goff *et al*, 1994) and is characterized by a highly aggressive biologic behaviour (Carcangiu and Chambers, 1992; Goff *et al*, 1994; Sherman *et al*, 1992). In contrast to ovarian cancer, however, it is a chemoresistant disease from onset with responses to combined cisplatinum-based chemotherapy in the order of 20% (Levenback *et al*, 1992; Nicklin and Copeland, 1996). The survival rate is dismal, even when USPC is only a component of the histologically more common endometrioid adenocarcinoma (Carcangiu and Chambers, 1992, 1995). The overall 5-year survival is 30±9% for all stages and the recurrence rate after surgery is extremely high (50–80%). Novel therapeutic strategies and a deeper molecular understanding of host interactions with possible carcinogenic factors are desperately needed.

To date, with the exception of melanoma (Castelli *et al*, 1996; Cox *et al*, 1994), there is limited information on the intrinsic immunogenicity or the identity and density of antigenic peptides and CTL epitopes presented by the majority of spontaneously arising human tumours. However, in the last few years several reports have shown that multiple epitopes can be recognized by T cells on several human tumours (i.e. ovarian, pancreatic, breast and lung) (Joannides *et al*, 1993; Peoples *et al*, 1995; Yoshino *et al*, 1994). Therefore, an alternative strategy for effective vaccination of tumour patients may be the use of unfractionated tumour-derived antigens such as whole tumour cells, peptides or proteins isolated from tumour cells. In this regard, effective tumour immunity in several murine tumour models has been induced using professional antigen presenting cells (pAPC) such as dendritic cells (DC) pulsed with unfractionated tumour-derived antigens in the form of peptides (Nair *et al*, 1997a; Zitvogel *et al*, 1996), cell sonicates (Nair *et al*, 1997b), or messenger RNA (mRNA) (Boczkowski *et al*, 1996).

Studies performed by several groups including our own have recently established the key role played by dendritic cells (DC) in the immune system and provide a rationale for using DC as natural adjuvants for human immunotherapy (Santin *et al*, 1999a; Steinman, 1991; Young and Inaba, 1996). DCs are the most effective APC at activating naive T cells (Steinman, 1991; Young and Inaba, 1996), and recently the combination of GM-CSF and IL-4 has been shown to generate large numbers of DCs from peripheral blood monocyte precursors (Romani *et al*, 1994; Sallusto and Lanzavecchia, 1994). In this study, we have used autologous DCs pulsed with USPC lysate to induce a tumour-specific T cell response from three USPC patients. Here, using a completely autologous system, we report the *in vitro* induction of HLA class I-restricted CD8^+^ CTLs in patients with advanced USPC. In addition, using two colour flow cytometric analysis of intracellular cytokine expression at the single cell level, we show that a strongly polarized Type 1 pattern of cytokine expression was inducible in the tumour antigen-primed CD8^+^ T cells of two patients, and that this phenotype correlated with high cytotoxic activity. In contrast, tumour antigen-primed CD8^+^ T cells from patient 3 showed minimal tumour-specific cytotoxicity, and exhibited a strong Type 2 bias in cytokine expression. These results may have important implications for treatment with active or adoptive immunotherapy of chemotherapy and radiotherapy resistant USPC.

## MATERIALS AND METHODS

### Patients

Three patients who had undergone total abdominal hysterectomy and regional lymph node sampling for invasive USPC provided tumour tissue and peripheral blood mononuclear cells (PBMC). Specimens were obtained at the time of surgery through the Gynecologic Oncology Department and the Pathology Department of the University of Arkansas for Medical Sciences (UAMS), Little Rock, Arkansas, under approval of the Institutional Review Board. Patient 1 had stage IIIA endometrial cancer and was aged 34 years, while patients 2 and 3 had stage IIIC adenocarcinoma and were aged 52 and 53 years, respectively. Patients did not receive any form of therapy prior to surgery.

### Tumour cell lines

The natural killer (NK) sensitive target K562 (a human erythroleukaemia cell line) was purchased from American Type Culture Collection and was maintained at 37°C, 5% CO_2_ in complete medium (CM) containing RPMI 1640 (Gibco-BRL, Grand Island, NY, USA), 10% foetal bovine serum (FBS, Gemini Bioproducts, Calabasas, CA, USA). Fresh autologous tumour samples were obtained from surgical specimens from all patients and were reduced to single cell suspensions under sterile conditions at room temperature, as previously described (Santin *et al*, 1996). Fresh tumour cell lines were maintained initially in RPMI 1640, supplemented with 15% autologous ascites fluid, and thereafter when established with 10% FBS at 37°C, 5% CO_2_. Briefly, single cell suspensions were obtained by processing solid tumour samples under sterile conditions at room temperature. Viable tumour tissue was mechanically minced in RPMI 1640 to portions no larger than 1–3 mm^3^ and washed twice with RPMI 1640. The portions of minced tumour were then placed into 250 ml trypsinizing flasks containing 30 ml of enzyme solution (0.14% Collagenase Type I (Sigma, St. Louis, MO, USA) and 0.01% DNAse (Sigma, 2000 KU mg^−1^)) in RPMI 1640, and incubated on a magnetic stirring apparatus overnight at 4°C. Enzymatically dissociated tumour was then filtered through 150 μm nylon mesh to generate a single cell suspension. The resultant cell suspension was then washed twice in RPMI 1640 plus 15% autologous ascites and thereafter seeded in T-75 or T-150 tissue cultures flasks (Corning, Costar Corp., Cambridge, MA, USA). The percentage of tumour cells used for tumour lysate applied to the DC or used as target cells in cytotoxicity assays (see below) was determined by cytokeratin expression using immunohistochemical techniques. All cell lines evaluated contained >99% tumour cells.

### HLA phenotypic analysis of CD8^+^ cultures

HLA class I typing of purified CD8^+^ cultures was performed by standard lymphocytotoxicity tests (Hopkins, 1990) in the tissue typing laboratory of the bone marrow transplantation and blood transfusion service at UAMS.

### Preparation of tumour lysate

5–10×10^6^ autologous tumour cells cultured in RPMI 1640 15% autologous ascites were washed twice with phosphate-buffered saline (PBS, pH 7.4) and harvested by scraping. Cells were lysed by three to four freeze cycles (in liquid nitrogen) and thaw cycles (room temperature). Lysis was monitored by light microscopy. Larger particles were removed by centrifugation (10 min, 400 **g**), supernatants were passed through a 0.2-μm filter, and stored at −80°C until use.

### Isolation of PBMC and generation of dendritic cells

PBMCs were separated from heparinized venous blood by Ficoll-Hypaque (Sigma) density gradient centrifugation and either cryopreserved in RPMI 1640 (Gibco-BRL) plus 10% DMSO and 30% autologous plasma, or immediately used for DC generation. Briefly, PBMC obtained from 42 ml of peripheral blood were placed into 6-well culture plates (Costar, Cambridge, MA, USA) in AIM-V medium (Gibco-BRL) at 0.5-1×10^7^/3 ml per well. After 2 h at 37°C, nonadherent cells were removed, and the adherent cells were cultured at 37°C in a humidified 5% CO_2_/95% air incubator, in medium supplemented with recombinant human GM-CSF ((800 U ml^−1^), Immunex, Seattle, WA, USA) and IL-4 ((1000 U ml^−1^) Genzyme, Cambridge, MA, USA) (Romani *et al*, 1994). Every 2 days, 1 ml of spent medium was replaced by 1.5 ml of fresh medium containing 1600 U ml^−1^ GM-CSF and 1000 U ml^−1^ IL-4, to yield final concentrations of 800 U ml^−1^ and 500 U ml^−1^, respectively (Romani *et al*, 1994). After 6 or 7 days of culture, DC were harvested for pulsing with tumour lysate as described below. The DC purity (i.e. cells strongly expressing HLA-DR^+^, CD86^+^, CD40^+^, and CD14^-^) ranged from 58 to 86% of the total cell population with a mean of 69±12%, as previously characterized by our laboratory (Santin *et al*, 1999b). No significant differences were noted in the expression of HLA-DR or costimulatory molecules (i.e., CD86, CD40), among DC cultures derived from the three patients (data not shown).

### DC pulsing

Following culture, DC were washed twice in AIM-V and added to 50 ml polypropylene tubes (Falcon, Oxnard, CA, USA). The cationic lipid DOTAP (Boehringer Mannheim, Indianapolis, IN, USA) was used to deliver the total cell extract into cells. Five hundred microlitres of total cell extract derived from 5–10×10^6^ tumour cells in AIM-V and DOTAP (125 μg in 500 μl of AIM-V) were mixed in 12×75 mm polystyrene tubes at room temperature for 20 min. The complex was added to the DC in a total volume of 2–5 ml of AIM-V and incubated at 37°C with occasional agitation for 3 h. The cells were washed twice with PBS and resuspended in AIM-V as described below.

### *In vitro* generation of tumour-specific CTLs

Fresh or cryopreserved responder PBMC were washed and resuspended in AIM-V at 10–20×10^6^ cells per well in 6-well culture plates (Costar) with tumour lysate-pulsed autologous DC (ratios from 20 : 1 to 30 : 1 responder PBMC: DC). The cultures were supplemented with recombinant human GM-CSF (500 U ml^−1^) and recombinant human IL-2 (10 U ml^−1^ Aldesleukin, Chiron Therapeutics, Emeryville, CA, USA) and incubated at 37°C. Human rIL-2 (10 U ml^−1^) was added to the cultures thereafter every 3-4 days. At day 21, CD8^+^ cells were separated from the bulk cultures by positive selection with CD8-Dynabeads (Dynal Inc., Lake Success, NY, USA) and further expanded in number for 7–10 days using autologous or allogeneic irradiated PBL (5000 cGy) (1×10^6^ cells per well) as feeder cells and anti-CD3 monoclonal antibody (Ortho Pharmaceutical Corp, Raritan, NJ, USA) (0.2 μg ml^−1^) plus 5% autologous plasma in 24-well plates (Costar) before being assayed for CTL activity. As negative control targets, autologous lymphoblasts were prepared by 3-day stimulation with Con-A (Gibco-BRL; 1 μg ml^−1^) in RPMI-1640 plus IL-2 (25 U ml^−1^), while EBV-transformed autologous lymphoblastoid B-cell lines (LCL) were established by coculture of PBMCs with EBV-containing supernatant from the B95.8 cell line in the presence of 1 μg ml^−1^ cyclosporin A (Sandoz, Camberley, UK) and were maintained in RPMI supplemented with 10% human AB serum (Gemini Bioproducts).

### Cytotoxic activity

A 6-h chromium (^51^Cr) release assay was performed as previously described (Nazaruk *et al*, 1998) to measure the cytotoxic reactivity of DC-tumour lysate stimulated CD8^+^ T lymphocytes. In addition to autologous tumour cells, K562 cells were used as a target for the detection of NK activity. Con-A activated peripheral blood lymphocytes and/or EBV-transformed LCL were used as autologous control targets. To determine the structures on the effector and target cells involved in lysis, monoclonal antibodies (MAbs) were used to block cytotoxicity. Effector cells were preincubated for 30 min at room temperature with MAbs which recognize human anti-CD11a/LFA-1 (10 μg ml^−1^) (PharMingen, San Diego, CA, USA) and its isotype control (IgG1k mAb isotype standard anti-TNP (10 μg ml^−1^) (PharMingen)). ^51^Cr-labelled tumour targets were preincubated with MAbs specific for monomorphic HLA class I (W6/32) (50 μg ml^−1^). The effector cells and ^51^Cr-labeled targets were then incubated in a final volume of 200 μl per microwell at 37°C with 6% CO_2_.

### Phenotypic analysis of T cells and autologous tumour cells

Enriched cultures of CD8^+^ T cells were phenotyped at the time of the first cytotoxicity assay and thereafter in order to correlate cytolytic specificity with a particular lymphoid subset. Flow cytometry was performed using MAbs directly conjugated against the following human leukocyte antigens: Leu-4 (CD3, pan T cells); Leu-3 (CD4, T helper/inducer); Leu-2a (CD8, T cytotoxic/suppressor); Leu-19 (CD56, NK/K cells); anti-CD16; Tac (CD25, the IL-2R); anti-HLA-DR (L-243); anti TcR-α/β or TcR-γ/δ (all Becton Dickinson, San Jose, CA, USA) and analyzed on a FACScan (Becton Dickinson). Autologous tumour cells were analyzed for MHC class I expression as previously described (Santin *et al*, 1996). Briefly, tumour cells were harvested with 0.25% Trypsin in HBS (Gibco-BRL) and washed once in CM. Cell suspensions were counted and distributed into 12×75 mm tubes at 5×10^5^ cells per tube. Mouse monoclonal antibodies were diluted in cold assay buffer (PBS, pH 7.2, supplemented with 0.1% FCS) and added in a 50-μl volume. A mouse IgG preparation (mAb IgG2a; Becton Dickinson) was used as negative control. Analysis was conducted with FACScan utilizing Cell Quest software (Becton Dickinson).

### Flow cytometric analysis of intracellular cytokines

This protocol is adapted from that described by Openshaw *et al* (1995). Flow cytometric analysis of intracellular cytokine expression was conducted essentially as previously described (Santin *et al*, 1999a). CD8^+^ T cells were tested at about 6 weeks after priming, after resting for 14 days after last antigen stimulation. Briefly, T cells (7.5×10^5^ ml^−1^) were incubated at 37°C for 6 h in AIM-V 5% autologous plasma plus 50 ng ml^−1^ PMA and 500 ng ml^−1^ ionomycin. Ten μg ml^−1^ Brefeldin A was added for the final 3 h of incubation. Controls, (non-activated cultures) were incubated in the presence of Brefeldin A only. The cells were harvested, washed and fixed with 2% paraformaldehyde in PBS for 20 min at room temperature, after which they were washed and stored overnight in PBS at 4°C. For intracellular staining, the cells were washed and permeabilized by incubation in PBS plus 1% BSA and 0.5% saponin (S-7900, Sigma) for 10 min at room temperature. Activated and control cells were stained with FITC-anti-IFN-γ, and PE-anti-IL-4, and isotype-matched controls (FITC-anti-Igγ2a and PE-anti-Igγ1) from Becton-Dickinson. After staining, cells were washed twice with PBS plus 1% BSA and 0.5% saponin, once with PBS plus 0.5% BSA, and fixed a second time with 2% paraformaldehyde in PBS. Analysis was conducted with a FACScan utilizing Cell Quest software (Becton Dickinson).

## RESULTS

### HLA typing

The peripheral blood mononuclear cells from the USPC patients manifested the following haplotypes: PT 1; HLA A24, A66, B7, B44, Cw7, Cw16, Bw4, Bw6. PT 2; HLA A3, A23, B51, B58, Cw6, Cw5, Bw4. PT 3; HLA A23, B49, Cw7, Bw4.

### HLA class I expression by autologous tumour cells

**Figure 1:**
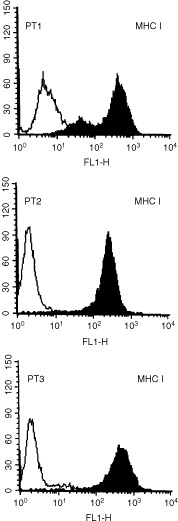
Expression of MHC Class I molecules on USPC cells. Surface antigen expression was measured by FACS as described under Materials and methods. A representative experiment is shown. Open profile: isotype control; solid profile: anti-MHC class I MAbs.

MHC class I expression was evaluated by FACS analysis on the tumour cell lines established from all three patients. As can be seen in Figure 1

, all tumour cell lines expressed MHC class I molecules. In addition, IFN-γ exposure was able to induce a significant upregulation of HLA class I expression in all three cell lines (data not shown).

### Tumour-specific CD8^+^ cytotoxic T cell responses

**Figure 2:**
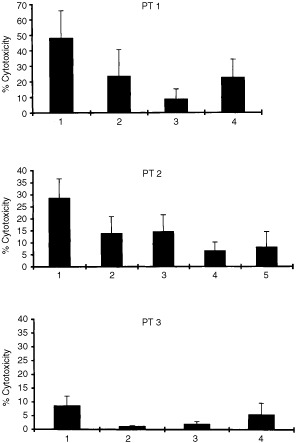
Tumour specific CD8^+^ CTL responses induced by tumour peptide-pulsed DCs in patients with USPC, measured in a 6 h ^51^Cr-release assay. Percentage lysis (± standard deviation) at a 20 : 1 effector/target cell ratio is shown. Anti-HLA class I blocking antibody (W6/32) was used at 50 μg ml^−1^ while anti-CD11a/LFA-1 was used at 10 μg ml^−1^. Patient 1; 1, Autologous tumour; 2, Autologous tumour+W6/32 anti-HLA class I MAb; 3, LCL control; 4, K562. Patient 2; 1, Autologous tumour; 2, Autologous tumour+W6/32 anti-class I MAb; 3, Autologous tumour+anti-LFA-1 MAb; 4, LCL control; 5, K562. Patient 3; 1, Autologous tumour; 2, Autologous tumour+W6/32 anti-HLA Class I MAb; 3, LCL control; 4, K562. Values are presented as the mean of at least three independently primed T cell cultures.

Cytotoxicity assays were conducted with purified CD8^+^ T cells at a minimum of 4 weeks after stimulation of T cell cultures with tumour lysate-pulsed DC. Results are presented as the mean values from at least three independently primed CD8^+^ T cell cultures from each patient. As shown in Figure 2

, cytotoxicity against autologous tumour cells was demonstrated for patients 1 and 2, at an effector:target ratio 20 : 1. For these patients, tumour specific cytotoxicity was significantly inhibited by blocking MAb against HLA class I (significant at *P*<0.001 for patient 1, and *P*<0.05 for patient 2, Student's matched-pairs *t*-test). Lysis of NK-sensitive K562 cells was also observed, notably for the CD8^+^ T cells from patient 1. However, lysis of autologous tumour cells was significantly higher than lysis of K-562 cells (*P*<0.05 for both patients 1 and 2). Collectively, these results suggest that specific, HLA class I-restricted cytotoxicity against autologous tumour antigens is a major component of the CD8^+^ T cell response following stimulation with tumour lysate-pulsed DC, although an NK-like CD8^+^ cytotoxic T cell response is also detected from patient 1. In patient 2, we also show that anti-CD11a (LFA-1) MAb were able to block tumour lysis, the range of inhibition being from 50 to 65% (Figure 2). This observation suggests that the CD11a-CD54 adhesion pathway may play an important role in effective CD8^+^ T cell lysis of USPC target cells. In contrast to the cytotoxic responses generated from patients 1 and 2, we were able to generate only a very low level of T cell mediated cytotoxicity from a single DC-primed CD8^+^ T cell culture from patient 3 (Figure 2). Two further attempts at priming tumour-specific CD8^+^ cytotoxic T cell responses from patient 3 were unsuccessful. For all three patients, minimal levels of cytotoxicity against autologous Con A-stimulated lymphoblasts (data not shown) or EBV-transformed LCL were observed.

### Phenotypic analysis of CD8^+^ T cells

**Figure 3:**
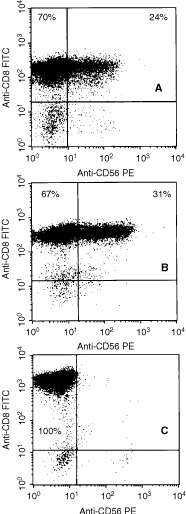
Two-colour flow cytometric analysis of CD56 expression by ovarian tumour-specific CD8^+^ T cells. T cells were phenotyped at the time of first cytotoxicity and thereafter as described in the Materials and methods. A representative experiment for each patient is shown. (**A**, **B** and **C**) Patients 1, 2 and 3, respectively.

Flow cytometric analysis was used to determine the phenotype of the populations of tumour-stimulated CD8^+^ T-cells derived from the three patients. All the cells were CD3/CD8^+^ and CD4^−^, with a variable proportion of CD56-antigen positive cells. CD8^+^ T cells were also TCR-αβ+ (94–98%), TCR-γδ+ (2–5%), CD25+, HLA-DR+ and CD16− (data not shown). Co-expression of CD56 on CD8^+^ T lymphocytes was further analyzed by two colour immunofluorescence (Figure 3

). CD8^+^ T lymphocytes from patients 1 and 2, but not patient 3 (Figure 3), co-expressed the CD56 surface antigen during culture (range from 15 to 52%).

### Intracellular cytokine expression by tumour-specific T cells

**Figure 4:**
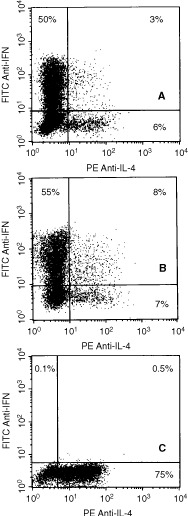
Two-colour flow cytometric analysis of intracellular IFN-γ and IL-4 expression by tumour specific CD8^+^ T cells. T cells were tested at about 6 weeks after priming, after resting for 14 days after the last antigen stimulation prior to activation by PMA and ionomycin. (**A**, **B** and **C**) Patients 1, 2 and 3, respectively. A representative experiment for each patient is shown.

To evaluate whether cytokine expression from tumour-stimulated CD8^+^ T cells segregated in discrete IFN-γ+/IL-4− and IFN-γ-/IL-4+ subsets we took advantage of recently developed flow cytometric techniques for the detection of intracellular cytokine expression at the single cell level. Two colour flow cytometric analysis of intracellular IFN-γ and IL-4 expression by CTLs was performed after 6 weeks of culture. As shown in Figure 4

, a large majority of CD8^+^ T cells from patients 1 and 2 expressed intracellular IFN-γ but not IL-4. In contrast, CD8^+^ T cells from patient 3 showed a striking dominance of IL-4 expressors (Figure 4) and less than 1% expressed IFN-γ. Similar results were obtained in several repetitive analyses for all patients. Unactivated (i.e. resting) CD8^+^ T cells from all three patients failed to stain for IFN-γ or IL-4. Similarly, FITC-anti-IgG2a and PE-anti-IgG1 isotype controls did not stain either activated or unactivated CD8^+^ T cells (data not shown).

## DISCUSSION

Tumour-specific CTLs recognizing multiple epitopes on a variety of epithelial tumours have recently been generated *in vitro* from the peripheral blood of cancer patients (Joannides *et al*, 1993; Peoples *et al*, 1995; Yoshino *et al*, 1994). Unfortunately, the possible immunization of cancer patients using defined tumour antigens is limited at present to a handful of human tumour types in which candidates for tumour rejection antigens have been identified. In an attempt to overcome these major limitations, several investigators have recently reported induction of effective tumour immunity using DCs pulsed with unfractionated tumour-derived materials, acid eluted peptides or messenger RNA (mRNA) (Boczkowski *et al*, 1996; Nair *et al*, 1997a,b; Zitvogel *et al*, 1996). Hence, DC vaccine treatment might be extended to patients with a variety of solid tumours, provided that *in vitro* confirmation of tumour-specific CTL activity has been achieved.

In this study, we show that strong CD8^+^ cytotoxic T cell responses against autologous tumour cells can be induced in USPC patients following stimulation of PBL with tumour lysate-pulsed DC. Although CD8^+^ cytotoxic T cells stimulated in this fashion showed some lytic activity against NK-sensitive K562 cells, much higher levels of cytotoxicity against autologous tumour cells were observed from patients 1 and 2. Furthermore, tumour-specific cytotoxicity was significantly inhibited by blocking MAb specific for HLA class I, indicating that a classical HLA-restricted CD8^+^ cytotoxic T cell response against tumour antigens was a major component of the response. In addition, CD16, a hallmark of NK cells, was not expressed by DC-stimulated CD8^+^ T cells. Phenotypic analysis revealed a significant CD56^+^ subpopulation within the CD8^+^ CTL from patients 1 and 2, but not from patient 3. This is in agreement with our previous results on CD8^+^ CTL populations stimulated by HPV16/18 full length E7-pulsed DC in cervical cancer patients (Santin *et al*, 1999a), as well as the results of others (Hilders *et al*, 1994; Pittet *et al*, 2000), where CD56 expression by CD8^+^ CTL was shown to correlate with cytotoxic activity against autologous tumour cells. In addition, the highest level of CD56 expression was observed with the CD8^+^ CTL from patient 2, which showed anti-HLA class I-blockable tumour lysis, but negligible killing of K562 or other control targets. From these observations, we conclude that CD56 expression by CD8^+^ CTL does not correlate with NK activity. Autologous LCL or Con-A activated blasts were not killed by tumour specific CTLs, indicating that while these CTLs were highly cytolytic for autologous tumour cells, they failed to kill autologous normal cells.

T cell-mediated protection from viral infection as well as control of tumours is thought to be promoted by Type 1 cytokine responses and impaired by Type 2 cytokine responses (for review see Romagnani, 1992). In general, Type 1 T cells (CD4 or CD8) express IL-2, IFN-γ, and TNFα/β, and are cytotoxic, whereas Type 2 T cells express IL-4, IL-5, IL-6, IL-10 and IL13, provide efficient help for B cell activation, and are non-cytotoxic. Consistent with this view, recent studies have showed significant dysfunction of Type 1 T cell responses in patients with cancer, suggesting that progression of disease may be associated with a preferential Type 2 T cell response (Ghosh *et al*, 1995; Romagnani, 1992; Tsukui *et al*, 1996). Selection of T cell phenotype at the time of the interaction with Ag-pulsed DC is known to be determined by a number of factors including (1) the cytokine milieu at priming, with IL-12 favouring Type 1 and IL-4 favouring Type 2 responses, (2) the nature and the intensity of TCR-mediated activation signals, (3) the percentage of naive and primed T cells present at the time of antigen exposure and (4) the costimulatory signals provided by surface molecules expresssed on APC as well as their level of expression (for review see Coffman *et al*, 1999). Two colour flow cytometric analysis of intracellular IFN-γ and IL-4 expression by CD8^+^ T cells demonstrated that tumour-specific T cells from USPC patients 1 and 2 showed a major Type 1 bias in cytokine expression. Indeed, a large majority of cytokine expressing T cells showed IFN-γ expression while a second minor subset contained only IL-4 and a third subset contained both (Figure 4). Patient 3 showed a low but reproducible level of cytotoxicity, ranging from 6 to 13% from only the first DC-primed CD8^+^ T cells. Two successive attempts of priming conducted with PBMC obtained from the patient at the time of histologically confirmed gross tumour progression in the lymphatic system (lumbo-aortic lymph nodes) were unsuccessful. The reasons why tumour lysate pulsed DC stimulated CD8^+^ T cells from these primings from patient 3 were not able to generate a cytotoxic response against autologous tumour cells are open to speculation. However, it is notable that a striking dominance of IL-4 over IFN-γ expressing CD8^+^ T cells from patient 3 was reproducibly detected by flow cytometry. We consider it probable that a strong Type 2 bias in the CD8^+^ T cell response accounts for the lack of cytotoxic effector function from this patient. Supporting this view, CD8^+^ T cells isolated during these ineffective primings secreted large amounts of IL-10 as detected by ELISA when stimulated by solid phase OKT3 (data not shown). A more difficult question is why patient 3 should respond in this manner. It is possible that the Type 2 bias in T cell responses, which was also observed for CD4^+^ T cells from patients 3 (not shown), may be related to the progressive nature of the disease. At the time of the first DC priming, which generated a limited cytotoxic response, the patient had gross endometrial disease, but limited metastatic spread, whereas later tumour lysate-pulsed DC primings, which consistently induced non-cytotoxic Type 2 CD8^+^ T cell responses, were attempted when the tumour had heavily involved the regional pelvic and lumbo-aortic lymph nodes. Extensive lymph node metastasis may thus be associated with tumour specific tolerance (Santin, 2000).

Taken together, the findings of this study show that patients harbouring locally advanced USPC with limited metastatic spread to the secondary lymphoid organs can elicit a powerful cytotoxic T cell response against their autologous tumour *in vivo* following tumour lysate-pulsed-DC stimulation. However, evidence of tumour specific tolerance in some patients may impose a significant barrier to DC-based immunotherapy. In this group of patients, attempts to alter established Th2 responses with the use of strongly polarizing Th1 agents such as IL-12, IFN-γ, IFN-α, anti-IL-4 and anti-IL-10 MAbs (Coffman *et al*, 1999) might therefore have considerable therapeutic implications.
